# Introduction to the Special Issue of *Plants* on “The Application of Spectral Techniques in Agriculture and Forestry”

**DOI:** 10.3390/plants13182632

**Published:** 2024-09-20

**Authors:** Youzhen Xiang

**Affiliations:** 1 Key Laboratory of Agricultural Soil and Water Engineering in Arid and Semiarid Areas of Ministry of Education, Northwest A&F University, Yangling 712100, China; youzhenxiang@nwsuaf.edu.cn; 2 Institute of Water-Saving Agriculture in Arid Areas of China, Northwest A&F University, Yangling 712100, China

This Special Issue, titled “Applications of Spectral Technology in Agriculture and Forestry”, presents a collection of cutting-edge research findings exploring various applications of spectral analysis in agricultural and forestry environments. The papers in this issue collectively examine the use of advanced spectral methods across key domains, including crop health monitoring, disease detection, forest parameter estimation, soil quality assessment, water stress analysis, and nutrient management. These studies not only highlight advances in their respective fields but also reveal the complex interplay between spectral technologies, machine learning, and sustainable resource management in agricultural ecosystems. Through the research presented, this Special Issue showcases an evolving paradigm where precision agriculture and forestry practices increasingly rely on sophisticated spectral data analysis for information acquisition and decision optimization. This Special Issue compiles research from around the world, covering diverse applications of spectral technologies in agriculture and forestry across different climates, ecosystems, and crop types. The twelve papers included demonstrate the broad applicability of these technologies in varying geographical regions and crops, emphasizing the efforts of scientists from multiple countries, including regions such as Europe and Asia, to promote precision agriculture and forestry practices. The following is an overview of each paper, providing insights into how they collectively advance the development of precision agriculture and forestry.

A common theme across these studies is the use of advanced spectral indices and remote sensing techniques to monitor various physiological parameters of plants. For example, Liu et al. (2024) [1] proposed a novel spectral index designed to overcome the angular effects on the estimation of the leaf area index (LAI) in winter rapeseed. Their method utilizes multi-angle hyperspectral data to test the stability of 16 traditional vegetation indices (VIs) in monitoring LAI from different observation angles. The study found that the OPIVI index exhibited the highest correlation in LAI estimation, providing valuable guidance for the selection of vegetation indices in future UAV and satellite applications. Shi et al. (2024) [2] focused on using hyperspectral data to monitor chlorophyll content in potato crops, demonstrating how differential transformations of spectral indices can effectively estimate chlorophyll levels. They constructed several machine learning models, including Support Vector Machine (SVM), Random Forest (RF), and Backpropagation Neural Network (BPNN) models, to predict potato chlorophyll content, highlighting the versatility of hyperspectral data in monitoring different physiological parameters. Both studies suggest that combining multi-angle and differential spectral indices with machine learning algorithms is an effective approach to capturing key physiological features of crop growth. Liu et al. (2024) and Shi et al. (2024) [1,2] provide complementary insights into the application of spectral data in precision agriculture, recommending the integration of various spectral indices with machine learning to construct a robust, non-destructive crop monitoring framework.

In the area of crop disease detection, spectral technology also plays a critical role. Danilov et al. (2024) [3] investigated the effects of disease development on the spectral characteristics of winter wheat varieties, revealing how disease severity alters wheat spectral reflectance, particularly in the near-infrared range. Their study demonstrated significant differences in the spectral characteristics of winter wheat varieties under disease influence, offering new possibilities for monitoring crop health and disease progression. In contrast, Zhou et al. (2024) [4] utilized an improved convolutional neural network (CNN) model, ShuffleNetV2, to identify maize leaf diseases. They introduced the SimAM attention mechanism to enhance the model’s accuracy in complex backgrounds. The results showed that the model achieved an accuracy of 98.40% on the maize leaf disease dataset, with a more compact model structure. Both studies underscore the importance of spectral data in disease detection, with the former focusing on near-infrared spectral monitoring of disease severity and the latter demonstrating the efficiency of deep learning models in disease identification.

The issue also discusses the application of spectral technology in forest parameter estimation and soil quality assessment. Ye et al. (2024) [5] provided a comprehensive review of L-band synthetic aperture radar (SAR) technology for forest canopy penetration and vertical structure parameter estimation, summarizing the application of L-band SAR in estimating forest height, moisture, and biomass. The study explored the challenges and future research directions of L-band SAR in forest resource management. Zhong et al. (2024) [6] studied how rice leaf spectra could be used to indirectly estimate heavy metal contamination in soil, utilizing a genetic algorithm-optimized partial least squares regression (GA-PLSR) model for soil quality monitoring. Despite focusing on different application areas, with Ye et al. concentrating on SAR technology in forestry and Zhong et al. (2024) [6] on spectral technology for agricultural soil monitoring, both studies emphasize the importance of remote sensing as an environmental assessment tool, showcasing how spectral technology can provide critical data for resource management.

Water stress analysis and nutrient management represent another field where spectral technology is making significant contributions. Wang et al. (2024) [7] investigated the impact of the time-lag effect between canopy temperature and atmospheric temperature on the accuracy of the Crop Water Stress Index (CWSI). They quantified the time-lag parameter for winter wheat and improved the predictive accuracy of CWSI using a genetic algorithm–support vector machine (GA-SVM) model. The study’s results showed that accounting for the time-lag effect effectively enhanced the correlation between CWSI and photosynthetic parameters, providing theoretical support for the application of thermal infrared remote sensing in crop water stress diagnostics. The study by Yang et al. (2024) [8] also focuses on crop water status diagnosis. They utilized UAV multispectral technology to estimate soybean leaf moisture through a comprehensive analysis of vegetation indices, canopy texture features, and randomly extracted texture indices. By employing Extreme Learning Machine (ELM), Extreme Gradient Boosting (XGBoost), and BPNN models, they achieved significant results, with the XGBoost model demonstrating the highest accuracy in leaf moisture monitoring. Similarly, Sun et al. (2024) [9] utilized spectral parameters to monitor nitrogen concentration in soybean leaves, finding the highest correlation between spectral parameters and nitrogen concentration in the upper leaves of the crop. They constructed several machine learning models, with the Random Forest (RF) model exhibiting the highest accuracy in estimating soybean leaf nitrogen concentration. Both studies highlight the integration of spectral data and machine learning to improve the accuracy of crop water and nutrient monitoring. Additionally, Nowack et al. (2024) [10] explored the use of UAV-mounted multispectral sensors to estimate vineyard water status under different pruning strategies, finding that red light and red-edge bands effectively predicted vine water status. This study further emphasizes the value of high-resolution multispectral imaging in crop water management. Zhang et al. (2024) [11] conducted field experiments to explore the effects of optimizing mulch type and nitrogen application rate on maize photosynthetic capacity, yield, and nitrogen use efficiency, discovering that using biodegradable plastic mulch combined with moderate nitrogen application significantly improved maize photosynthetic efficiency and yield. These studies highlight the potential of spectral technology in various nutrient conditions and farming practices.

Lastly, Bitella et al. (2024) [12] proposed a low-cost, near-ground platform for monitoring crop height and spatial distribution using ultrasonic sensors and spectral data, achieving precise monitoring of plant growth characteristics across different cropping systems. This research not only demonstrates the potential of low-cost remote sensing platforms in agriculture but also complements the studies by Liu et al. (2024) and Shi et al. (2024) [1,2], which utilize multi-angle and hyperspectral data to monitor crop growth, providing diverse technological pathways for precision agriculture.

In summary, the papers in this Special Issue provide a deeper understanding of the applications of spectral technology in precision agriculture and forestry management, expanding the research scope of this field. The topics covered, including crop health monitoring, disease detection, forest parameter estimation, soil quality assessment, water stress analysis, and nutrient management, reveal the diversity and practicality of spectral technology while emphasizing its crucial role in promoting sustainable development of agricultural ecosystems. These studies point to a rapidly evolving scientific frontier, where the deep integration of spectral data and machine learning techniques is set to become the core driving force for future precision agriculture and forestry development. The papers in this Special Issue draw on each other’s findings, employing multi-source data fusion, machine learning modeling, remote sensing, and hyperspectral analysis to establish a comprehensive and flexible analytical framework capable of real-time, accurate monitoring of crop and forest ecosystem dynamics. This framework provides both the theoretical foundation and practical pathways for addressing the increasingly complex challenges in agriculture and forestry. More importantly, these research findings not only provide a solid theoretical basis for the application of spectral analysis in multidisciplinary fields but also offer valuable guidance for scholars and practitioners in precision agriculture and forestry. Through this compilation, we witness the immense potential of spectral technology in data-driven decision making, sustainable resource management, and ecosystem health assessment, laying a solid foundation for future in-depth research and practical applications in related fields.
